# Preparation of Cellulose Nanofibers from Bagasse by Phosphoric Acid and Hydrogen Peroxide Enables Fibrillation via a Swelling, Hydrolysis, and Oxidation Cooperative Mechanism

**DOI:** 10.3390/nano10112227

**Published:** 2020-11-10

**Authors:** Jinlong Wang, Qi Wang, Yiting Wu, Feitian Bai, Haiqi Wang, Shurun Si, Yongfeng Lu, Xusheng Li, Shuangfei Wang

**Affiliations:** 1 School of Light Industrial and Food Engineering, Guangxi University, Nanning 530004, China; long05360525@163.com (J.W.); w1497417327@163.com (Q.W.); ww1031327514@163.com (Y.W.); baifeitian123@163.com (F.B.); m15305371443@163.com (H.W.); ssr9979@163.com (S.S.); lyf1102891715@163.com (Y.L.); wangsf@gxu.edu.cn (S.W.); 2 Guangxi Key Laboratory of Clean Pulp & Papermaking and Pollution Control, Nanning 530004, China

**Keywords:** cellulose nanofibers, lignocellulosic biomass, swelling, oxidation, hydrolysis

## Abstract

Due to the natural cellulose encapsulated in both lignin and hemicellulose matrices, as well as in plant cell walls with a compact and complex hierarchy, extracting cellulose nanofibers (CNFs) from lignocellulosic biomass is challenging. In this study, a sustainable high yield strategy with respect to other CNF preparations was developed. The cellulose was liberated from plant cell walls and fibrillated to a 7–22 nm thickness in one bath treatment with H_3_PO_4_ and H_2_O_2_ under mild conditions. The cellulose underwent swelling, the lignin underwent oxidative degradation, and the hemicellulose and a small amount of cellulose underwent acid hydrolysis. The CNFs’ width was about 12 nm, with high yields (93% and 50% based on cellulose and biomass, respectively), and a 64% crystallinity and good thermal stability were obtained from bagasse. The current work suggests a strategy with simplicity, mild conditions, and cost-effectiveness, which means that this method can contribute to sustainable development for the preparation of CNFs.

## 1. Introduction

Lignocellulosic biomass is considered the most abundant source of natural biopolymer on Earth, with a total annual output of 146 billion tons [1]. Therefore, obtaining sustainable resources from lignocellulosic biomass is important and has received increasing attention due to limited fossil-based resources [2,3]. Many nanoscale biopolymer building blocks, such as cellulose nanofibers (CNFs), with their high aspect ratio, low coefficient of thermal expansion, biocompatibility, biodegradability, and excellent mechanical and optical properties, have the potential to surpass fossil-based materials with respect to food packaging, biomedical applications, electronics, and high-performance materials [4,5,6,7,8,9].

Lignocellulose is a complex of lignin, hemicellulose, and cellulose present in plant cell walls [10,11]. Cellulose is a natural linear polymer composed of repeated β-D-glucopyranose units [12]. Cellulose chains easily aggregate into 3 nm thick elementary fibrils (EFs) that alternate between crystalline and amorphous regions. The EFs embedded in the hemicellulose matrix are further aggregated to form 10–25 nm thick microfibrils (MFs). MFs are embedded in the matrix composed of lignin and hemicellulose and then bond together in a spiral manner, thus weaving throughout plant cell walls to form a complex and compact hierarchical structure [13]. The extraction of CNFs from lignocellulosic biomass is challenging because the cellulose is encapsulated in both lignin and hemicellulose matrices, as well as in plant cell walls with a compact and complex hierarchy.

Traditionally, the required procedures, such as the removal of lignin, degradation of hemicellulose, and deconstruction of the plant cell wall, need to be achieved gradually, in order to extract CNFs from lignocellulosic biomass. For example, lignin is removed by both cooking using caustic soda, sulfate [14,15,16], or sulfite and bleaching with oxygen, hydrogen peroxide, or chlorine dioxide [17,18], while hemicellulose is hydrolyzed or oxidatively degraded by an acid, enzyme, or oxidant. Since the successful preparation of CNFs in 1983 [19], repeated mechanical treatment using high-pressure homogenization or microfluidization has remained a necessary procedure to produce uniformly sized CNFs [20]. In addition to the risk of clogging, a high energy consumption is required to cleave hydrogen bonds between the microfibrils and deconstruct the cell walls during the mechanical treatment [21]. These challenges limit the large-scale production of CNFs. Chemical pretreatments, such as carboxymethylation [12,22], sulfonation [23], oxidation [24], and hydrolysis [25], have been shown to be effective in improving the production efficiency, reducing the risk of clogging, and saving energy consumption for mechanical treatment [26]. TEMPO oxidation is considered a classical and effective method for preparing CNFs [12,22,27,28]. These methods are developed based on postprocessing, and multistep chemical-assisted treatment using multiple reagents is needed for the conversion from biomass to CNFs, which is prohibitively expensive for commercial applications. If all of the above necessary procedures for the extraction of CNFs from lignocellulosic biomass could be reached in a single bath via chemical-assisted treatment, this would lead to a more sustainable process.

In our previous research, cellulose nanofibers (CNFs) were successfully obtained from bamboo in one bath treatment with HNO_3_ and H_2_O_2_ [29]. The results showed that the synergetic action provided by HNO_3_ and H_2_O_2_, namely, acid hydrolysis and oxidation, enabled cellulose to be easily fibrillated during the following mechanical treatment. Phosphoric acid (H_3_PO_4_) is a relatively safe medium-strong acid (pK_a_ = 2.12) without strong corrosion and oxidation. H_3_PO_4_ hydrolysis has been used for the preparation of nanocellulose, such as cellulose particles [30], and cellulose nanocrystals [31,32,33,34]. H_3_PO_4_ has a strong ability to form hydrogen bonds with the hydroxyl group of cellulose, which is attributed to the fact that the four O atoms in the H_3_PO_4_ molecule all have strong electronegativity [35]. H_3_PO_4_ has been shown to be an effective swelling and dissolving agent for cellulose. If H_3_PO_4_ is adopted instead of HNO_3_ for the extraction of CNFs, it is beneficial to accelerate the oxidation and hydrolysis process.

In this work, through the usage of H_3_PO_4_ and H_2_O_2_, a cooperative ternary mechanism consisting of oxidation, swelling, and acid hydrolysis occurred. Therefore, cellulose encountered intercrystalline swelling in H_3_PO_4_ aqueous solution, lignin was oxidatively degraded by the OH+ produced by H_3_PO_5_, and hemicellulose and a small amount of cellulose were hydrolyzed by acid. The cellulose was liberated from plant cell walls and fibrillated. This method saves several independent dedicated procedures for the conversion from lignocellulosic biomass to CNFs. In addition, it has the potential of phosphoric acid in filtrate after treatment for the recycling and production of phosphate fertilizers [36,37,38]. Therefore, the CNF preparation strategy demonstrated here has the following characteristics: Simplicity; mild conditions; and cost-effectiveness. Considering this, it has the potential to enable the CNF industry to be more sustainable. A potential application of CNFs is an enhancement to the strength of recycled paper due to its tendency to self-assemble into films.

## 2. Materials and Methods

### 2.1. Chemicals and Raw Materials

Bagasse was provided by Guangxi Guitang (Group) Co., Ltd. (Guangxi, China). The bagasse was ground to a 40–60 mesh powder. Phosphoric acid (H_3_PO_4_, 85 wt. %), hydrogen peroxide (H_2_O_2_, 30 wt. %), and absolute ethanol (99.7 wt. %) were all analytically pure reagents purchased from Nanning Blue Sky Experimental Equipment Co., Ltd. (Nanning, China). Poly(ethyleneimine) solution (PEI, 50 wt. %) was purchased from Shanghai Macklin Biochemical Co., Ltd. (Shanghai, China).

### 2.2. Preparation of the Cellulose Nanofibers

**Figure 1:**
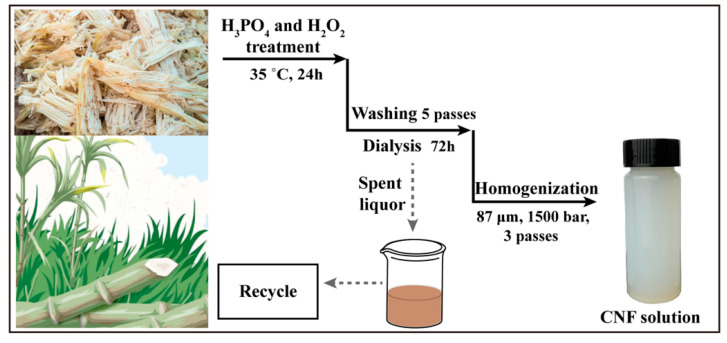
Schematic diagram of the extraction of cellulose nanofibers (CNFs) from bagasse by H_4_PO_3_ and H_2_O_2_ treatment.

A schematic diagram of the CNFs extracted from bagasse by the H_3_PO_4_ and H_2_O_2_ treatment is shown in Figure 1. Ten grams of bagasse powder was added to 300 mL H_3_PO_4_ aqueous solution (72 wt. %) and treated with 30 or 60 mmol/g H_2_O_2_ at 5 or 35 °C for 18, 24, 36, or 96 h. The suspension was mixed with four volumes of deionized water in a 1000 mL measuring cylinder at the end of the reaction, and the upper clear liquid was poured as the fibers settled, which was repeated five times. In order to remove the remaining acids, the suspension was dialyzed (72 h) until the suspension became neutral. The cellulose fibers were diluted to a concentration of 0.8 wt. % and homogenized using a microfluidization homogenizer (M-110EH30, MFIC, Westwood, MA, USA) with a pore size of 87 μm under 1500 bar. The homogenization procedure was repeated three times to obtain CNFs. 

### 2.3. Characterization

The CNFs employed for characterization were obtained from bagasse treated in the 72 wt. % H_3_PO_4_ solution with 60 mmol/g H_2_O_2_ at 35 °C for 24 h and then homogenized three times.

The chemical compositions of the bagasse and CNFs were determined according to the measurement method of the Association of Pulp and Paper Industry Technology (TAPPI) (Atlanta, GA, USA). The T203 OS-74 TAPPI standard was used to determine the content of cellulose and hemicellulose, while the T222 OS-83 TAPPI standard was used to determine the content of lignin.

Three grams of bagasse powder were weighed and dried in a drying oven at 105 °C for 4 h. The bagasse powder was cooled in a dryer and weighed. The moisture content of bagasse powder was calculated based on its mass before and after drying. Bagasse samples treated with phosphoric acid and hydrogen peroxide were freeze-dried by the AdVantage Plus EL-85 freeze-drying system (SP Scientific, Warminster, PA, USA) for 24 h and then weighed. The yield based on the initial amount of bagasse was calculated according to the following formula:

Yield = m_2_/m_1_ × 100%, (1)

where m_1_ and m_2_ represent the absolute dry mass of the bagasse and the treated sample, respectively.

According to the T203 OS-74 TAPPI standard, the cellulose content in the bagasse and the cellulose content in the treated sample were obtained. The cellulose yield based on the initial amount of cellulose in bagasse was calculated according to the following formula:

Cellulose yield = m_4_/m_3_ × 100%, (2)

where m_3_ and m_4_ represent the mass of the cellulose in the bagasse and in the treated sample, respectively.

The degree of polymerization (DP) of cellulose was calculated by the following formula:

DP^0.905^ = 0.75 × [954 × log(X) − 325], (3)

where X is the viscosity, which was measured according to the T 230 om-99 TAPPI standard method.

The phosphorus content of the freeze-dried cellulose was measured using an Agilent 720ES inductively coupled plasma optical emission spectrometer (ICP-OES) (Agilent, Palo Alto, CA, USA). The degree of phosphorus substitution (DS) was calculated by the following formula:

DS (%) = (m_5_/31) × 100%/(m_6_/162), (4)

where m_5_ and m_6_ represent the content of phosphorus and the mass of cellulose, respectively. Additionally, 31 and 162 were the atomic mass of the phosphorus and molecular mass of the cellulose monomer units, respectively.

The morphology and diameter of the CNFs were observed using transmission electron microscopy (TEM). The dialyzed cellulose fiber or homogenized CNF solution with a concentration of 0.0008 wt. % was deposited on a carbon-coated grid. After drying, it was negatively stained with 2% phosphotungstic acid in the dark for 20 min. TEM images were obtained on a JEM-1200EX instrument (JEOL, Tokyo, Japan) at an acceleration voltage of 200 kV. Nano Measurer software (Version 1.2, San Francisco, CA, USA) was used to determine the diameter of the nanofibrils after at least 100 measurements.

The CNF suspension was diluted to the concentration of 0.1% and 0.01% using deionized water, and then dispersed by ultrasonic treatment (20 min). The CNF samples for Atomic Force Microscopy (AFM) characterization were prepared according to the literature [39]. Mica pieces were immersed in PEI solution (3.3%). They were lightly rinsed with deionized water, and then immersed in 0.1% CNF solution for 1 min and left to dry naturally. Other samples were prepared by dropping one drop of 0.01% CNF suspension onto a mica sheet and then letting it dry naturally. The observation of the morphologies of the CNF samples was conducted using Hitachi Atomic Force Microscopy (5100N, Tokyo, Japan) in a tapping mode. Nano Measurer software (Version 1.2, San Francisco, CA, USA) was used to determine the width of the CNFs after at least 100 measurements. The height of CNFs was calculated by AFM 5000 II software (Hitachi, Tokyo, Japan).

The dialyzed cellulose fiber aqueous solution was diluted to a concentration of 1%, and the diluted solution was freeze-dried in the AdVantage Plus EL-85 freeze-drying system (SP Scientific, Warminster, PA, USA) for 24 h. The freeze-dried samples were then observed by a scanning electron microscope (SEM) (SU8220, Hitachi, Tokyo, Japan). All samples were sputter coated with gold before shooting.

Fourier transform infrared (FTIR) spectroscopy was recorded with a TENSOR II instrument (Bruker Technology, Ettlingen, Germany). The scanning range was 4000 to 400 cm^−1^, and the resolution was 4 cm^−1^.

X-ray diffraction (XRD) (MINFLEX 600, Tokyo, Japan) was used to determine the crystallinity of the lignocellulose and CNF powder samples. The crystallinity of the sample was calculated according to the following Equation [40,41]:

(5) $$\mathrm{CrI}\;\left(\%\right)=\frac{I_{200}-I_{\mathrm{am}}}{I_{200}}.$$

In this Equation, I_200_ represents the maximum intensity of the lattice diffraction peak at 2θ between 22° and 23°, and I_am_ represents the intensity scattered by the amorphous component in the sample, which was evaluated as the lowest intensity at 2θ between 18° and 19°.

The solid-state ^13^C cross-polarization magic angle spinning (^13^C CPMAS) nuclear magnetic resonance (NMR) spectra of the samples were acquired on an Agilent 600 MHz spectrometer (Agilent, Palo Alto, CA, USA).

Samples of bagasse and CNF powder were subjected to thermogravimetric analysis (TGA) in a synchronous thermal analyzer (NETZSCH STA 449F5, Selb, German). The test was conducted at a temperature increase rate of 10 °C·min^−1^ in a nitrogen atmosphere, and the temperature range was 30–600 °C.

## 3. Results and Discussion

### 3.1. Proposed Mechanism

**Figure 2:**
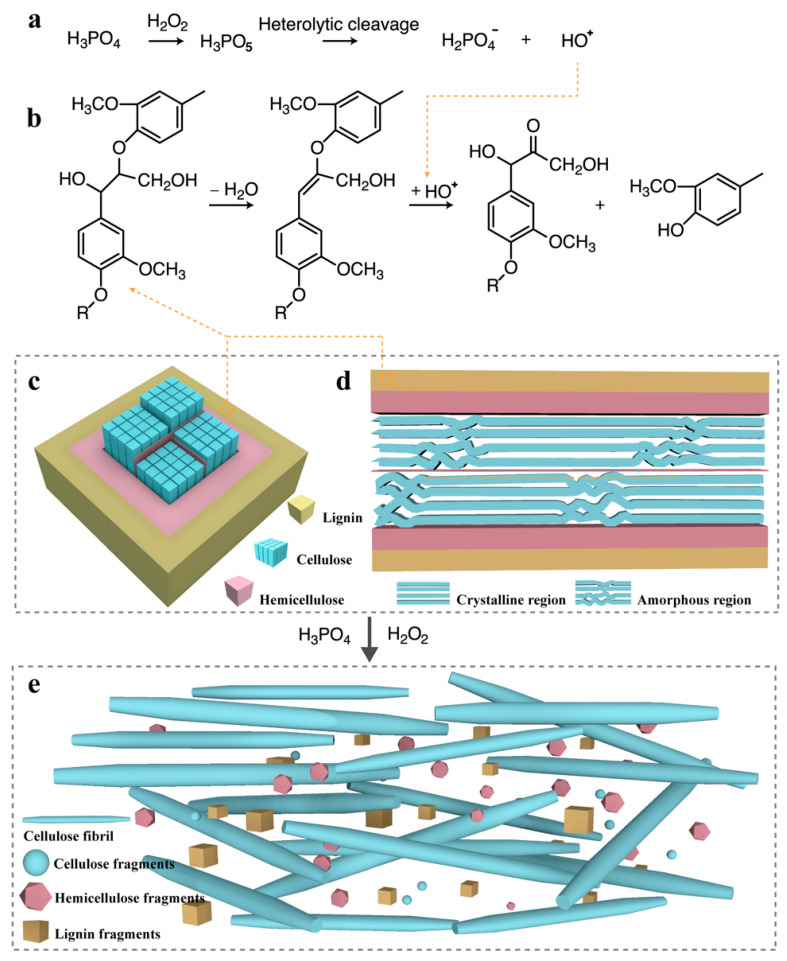
Schematic diagram of the microfibril structure in plant cell walls and the proposed mechanism for CNF preparation. (**a**) HO^+^ generated; (**b**) lignin oxidatively degraded by HO^+^; (**c**) cross-section of microfibrils; (**d**) longitudinal section of microfibrils; and (**e**) cellulose fibers swell and fibrillate in phosphoric acid solution.

Peroxyacid (H_3_PO_5_) may be produced by the reaction of phosphoric acid and H_2_O_2_ [42]. H_3_PO_5_ undergoes heterolytic cleavage in an acidic medium to produce HO^+^ (Figure 2a) [42]. HO^+^ is an electrophilic oxidizer. Therefore, the electron-rich functional groups (such as olefins, carbonyls, and aromatic ring structures) in lignin macromolecules were attacked by OH^+^, which caused the lignin to be oxidatively degraded into small molecules; at this point, the lignin was removed from the lignocellulosic biomass (Figure 2b) [43,44].

The polar hydroxyl groups readily adsorbed H_3_PO_4_ molecules with strong electronegativity. When lignocellulose is put into H_3_PO_4_ aqueous solution, H_3_PO_4_ molecules diffuse into amorphous and intercrystalline regions. The hydrogen bonds between cellulose are replaced by those between H_3_PO_4_ molecules and cellulose [45]. H_3_PO_4_ molecules filled the surrounding space, causing the cellulose to swell. The cellulose, which cannot be swollen by H_3_PO_4_, was separated into fibrillated fibers as intercrystalline swelling occurred (Figure 2e). H_3_PO_4_ molecules that formed hydrogen bonds with cellulose were replaced by water molecules during washing with water. In addition, some cellulose was dissolved in H_3_PO_4_ aqueous solution and then regenerated to produce cellulose II in the final product [46].

H_3_PO_4_ is a medium-strong acid (pK_a_ = 2.12) and tends to ionize, releasing H^+^, in aqueous solutions. The glycosidic bonds in cellulose have a low stability in acidic media and are prone to multiple hydrolysis processes. The accessible area of cellulose was hydrolyzed first, and the area with a lower accessibility was then hydrolyzed. Hemicellulose was degraded because the glycosidic bonds in hemicellulose can be cleaved by the water molecules in an acidic medium, which is similar to the acid hydrolysis of cellulose [47].

Through the use of concentrated H_3_PO_4_ and H_2_O_2_, a cooperative ternary mechanism consisting of oxidation, swelling, and acid hydrolysis occurred. Swelling occurred in areas accessible to cellulose by H_3_PO_4_ molecules. The lignin was removed by oxidative degradation. The hemicellulose was removed by acid hydrolysis. A small amount of cellulose was hydrolyzed, and the cellulose chains were cut off. The cellulose was separated into fibrillated fibers. A schematic diagram of the proposed mechanism for extracting CNFs from lignocellulose biomass by treatment with H_3_PO_4_ and H_2_O_2_ is shown in Figure 2.

### 3.2. Chemical Composition Analysis

Bagasse that was treated in the 72 wt. % H_3_PO_4_ aqueous solution with H_2_O_2_ dosages of 30 and 60 mmol/g at 5 and 35 °C for 18, 24, 36, and 96 h was studied by analyzing the chemical composition, yield, and removal ratio. The experimental data are shown in Table 1.

As shown in Table 1, as the treatment time was extended, the lignin removal percentages increased from 82.65% for 18 h (S.N.1) to 93.87% for 24 h (S.N.2) and then to 95.99% for 36 h (S.N.3). The removal ratios of lignin were 44.53% (S.N.4) and 93.87% (S.N.2) during treatment with the dosages of 30 and 60 mmol/g H_2_O_2_, respectively. This shows that the degree of lignin removal increased as the H_2_O_2_ dosage increased. The lignin removal ratios increased from 88.73% at 5 °C (S.N.5) to 93.87% at 35 °C (S.N.2) as the temperature increased. These phenomena showed that the H_2_O_2_ dosage, time, and temperature promoted lignin removal and that the lignin could be effectively removed during the treatment.

The ratios of hemicellulose removal increased as the time of the treatment increased from 96.32% after 18 h (S.N.1) to 98.80% after 24 h (S.N.2) and then to 99.95% after 36 h (S.N.3). The 91.18% hemicellulose removal ratios reached using the 30 mmol/g H_2_O_2_ treatment (S.N.4) were below the 98.80% hemicellulose removal ratios reached using the 60 mmol/g H_2_O_2_ (S.N.2), which may have been because the higher residual lignin content (20%) protected hemicellulose from acid hydrolysis using the 30 mmol/g H_2_O_2_ treatment (S.N.4). The percentage of hemicellulose removal during the treatment at 5 °C (70.69%, S.N.5) was far below that at 35 °C (91.18–99.95%, S.N.1–4). These experimental results showed that the H_2_O_2_ dosage, time, and temperature promoted hemicellulose removal. The hemicellulose could be effectively removed because the hemicellulose was fully exposed to the acidic medium due to the oxidative removal of lignin and the intercrystalline swelling of cellulose.

The cellulose yields based on the initial amounts of biomass and initial amount of cellulose in biomass decreased as the H_2_O_2_ dosage, time, and temperature increased. The yield of cellulose obtained from bagasse could reach 63.75% and 99.42% (S.N.5) based on biomass and cellulose, respectively. This was due to both the mild reaction conditions and high selectivity of HO^+^ delignification.

These phenomena indicated that the cooperative ternary mechanism of swelling, oxidation, and acid hydrolysis could efficiently remove hemicellulose and lignin while retaining cellulose.

### 3.3. Morphological Characterization

**Figure 3:**
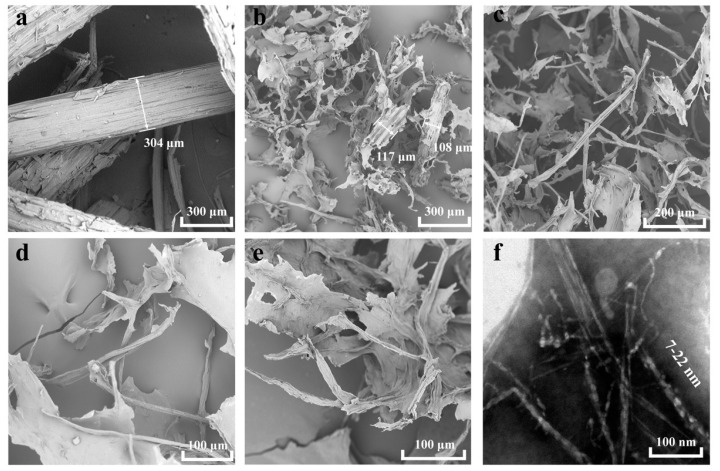
(**a**) Untreated bagasse fiber; (**b**–**e**) scanning electron microscope (SEM) images of bagasse treated with H_3_PO_4_ and H_2_O_2_ for 2, 6, 12, and 24 h, respectively (arrow length: fiber width); and (**f**) transmission electron microscopy (TEM) images of bagasse treated with H_3_PO_4_ and H_2_O_2_ for 24 h.

The morphologies of the bagasse powder and the fibers after treatment in 72 wt. % H_3_PO_4_ aqueous solution and using 60 mmol/g H_2_O_2_ at 35 °C for 2, 6, 12, and 24 h observed by SEM and TEM were compared, as shown in Figure 3. As can be seen from Figure 3a, bagasse powder with a particle width of approximately 300 µm and a compact surface structure was observed. Bagasse powder was deconstructed to produce a large number of lamellar fibers after treatment for 2 h (Figure 3b). The lamellar fibers were attributed to the fact that the separated fibers self-assembled into films. The fiber bundles had disappeared, the individual fibers and a large number of lamellar fibers were observed, and the fibrous cell walls collapsed after treatment for 6 h (Figure 3c), which was attributed to the removal of large amounts of lignin and hemicellulose. The individual fibers had almost transformed into lamellar fibers after treatment for 12 h or more (Figure 3d,e), which indicates that the plant cells had been deconstructed. The fiber-like morphologies may have resulted from the cross-linking of fibrils formed from the deconstruction of cell walls and subsequent self-assembly on this basis. Fibrils of a 7–22 nm width were observed (Figure 3f), which indicates that the cellulose was fibrillated during the treatment. The pearl-shaped substance attached to the surface of the CNF matrix was clearly observed. This may have been a cellulose microsphere that formed when the branching chains on the cellulose matrix were dissolved and then regenerated [48]. These experiments showed that the cellulose was liberated from the lignin and hemicellulose matrix and fibrillated via one bath treatment with a cooperative ternary mechanism of swelling, hydrolysis, and oxidation.

**Figure 4:**
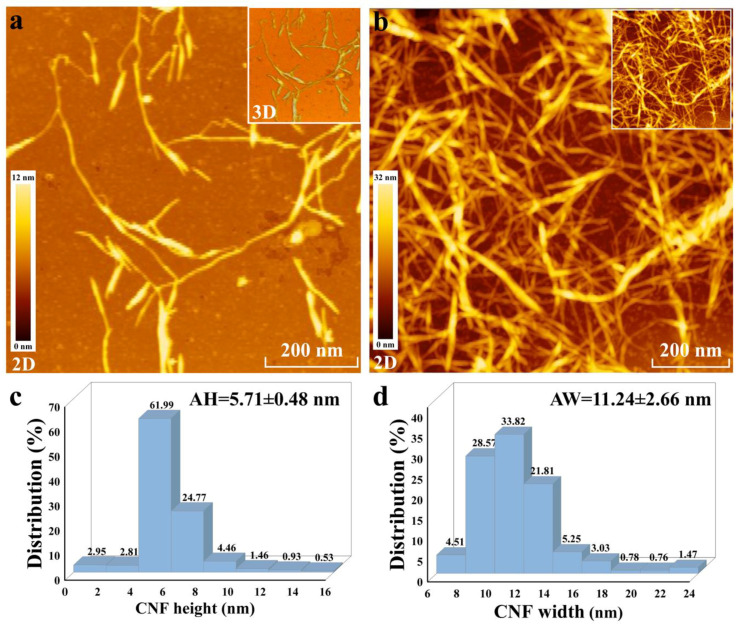
(**a**) Atomic Force Microscopy (AFM) image of CNF samples prepared based on the method by poly(ethyleneimine) solution (PEI) substrate adsorption; (**b**) AFM image of CNF samples prepared based on the method by dropping the suspension; and (**c**) height distribution and (**d**) width distribution of the CNFs measured based on AFM images (AH: average height and AW: average width).

The CNFs were obtained from the cellulose by the homogenization treatment. Their morphology was observed by AFM and TEM, their width was measured by Nano Measurer software, and their height was measured by AFM 5000 II software, which are all shown in Figure 4. As can be seen from Figure 4a, individual fibrils less than or equal to 12 nm in thickness were observed. Highly flocculated fibrils can be observed in Figure 4b. They stack up to a thickness of less than 32 nm. These fibrils look like branches that are not so soft. It may be that this unique treatment highlights the morphological characteristics of the cellulose crystalline zone skeleton. The skeleton structure is particularly obvious in AFM 3D images (Figure S1). The width of these CNFs is mainly distributed in the range of 9–13 nm, accounting for 84% of samples (Figure 4c), while the height of CNFs is mainly distributed in the range of 4–8 nm, accounting for 87% of samples (Figure 4d), indicating that CNFs have a high homogeneity. The average height and width of CNFs were 5.71 ± 0.48 nm and 11.24 ± 2.66 nm, respectively, and it was noteworthy that their height was much lower than their width. The obvious "flattening" phenomenon of CNFs was consistent with the research of Mattos et al. [39]. This was attributed to the adhesion and capillary forces during drying and the use of PEI further enhanced the effect of the adhesion [49]. CNFs also exhibited a clear skeleton structure in TEM images (Figure S2a). The average diameter of the CNFs measured based on TEM images was 12.19 ± 3.51 nm (Figure S2b). These phenomena indicated that the CNFs were prepared successfully.

The TEM diagram and XRD patterns of the bagasse treated in the 85 wt.% H_3_PO_4_ aqueous solution for 24 h are presented in Figures S3 and S4, respectively. Independent individual fibers were not observed in the XRD patterns (Figure S3). The characteristic absorption peaks at 2θ = 16.5° (110), 22.1° (200), and 34.7° (004) for cellulose I disappeared in the XRD spectra (Figure S4). These phenomena indicate that the intercrystalline swelling and intracrystalline swelling of cellulose are related to the concentration of H_3_PO_4_ solution [45]. The concentration range of H_3_PO_4_ aqueous solutions that have been shown to cause cellulose to be dissolved is above 77% [50], which should be avoided for the extraction of these CNFs.

These phenomena indicated that the CNFs were successfully extracted from untreated biomass in one bath pretreatment combined with homogeneous treatment. 

### 3.4. Characterization of Chemical and Physical Structures

**Figure 5:**
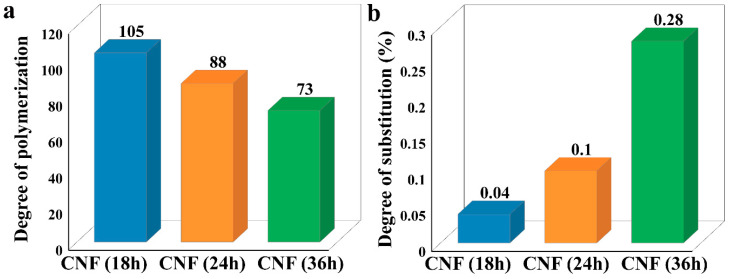
(**a**) Degree of polymerization (DP) of the cellulose in CNFs. (**b**) Degree of phosphorus substitution (DS) of the cellulose.

The DP of cellulose in the CNFs was tested, as shown in Figure 5a. As seen from Figure 5a, the DPs of cellulose in the CNFs from bagasse treated with H_3_PO_4_ and H_2_O_2_ for 18, 24, and 36 h were 105, 88, and 73, respectively. These experimental results showed that the DP of cellulose in the CNFs was low, which indicated that the cellulose macromolecular chain was cut because the cellulose in the accessible region was hydrolyzed by the treatment [51].

The DS of the CNFs were compared, as shown in Figure 5b. As can be seen from Figure 5b, as the treatment time increased, the DS of the CNFs increased from 0.04% after 18 h to 0.10% after 24 h and then increased to 0.28% after 36 h. This provides evidence that the CNFs had been esterified during the treatment. The esterification of cellulose by phosphoric acid may affect the surface charge of CNFs.

**Figure 6:**
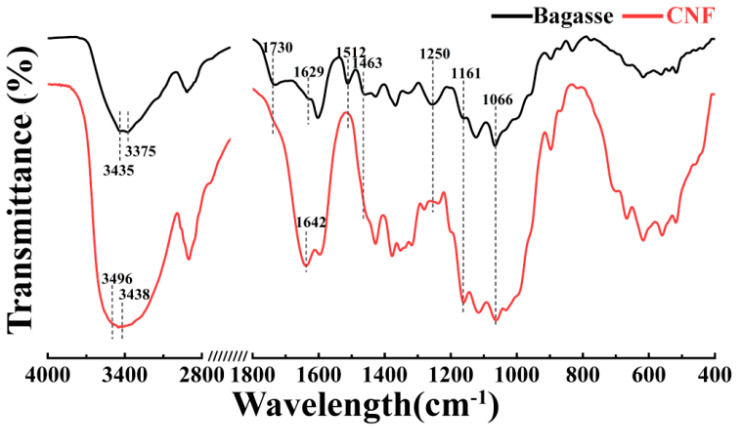
Fourier transform infrared (FTIR) spectra of the untreated bagasse and CNFs.

The FTIR spectra of the bagasse and CNFs are shown in Figure 6. As demonstrated in Figure 6, the characteristic peaks at 3435–3375 and 3496–3444 cm^−1^ (OH tensile vibrations of hydrogen bonding), 1161 cm^−1^ (C–O–C asymmetric stretching of cellulose), and 1066 cm^−1^ (C–O, C–C stretching vibration) of cellulose were clearly observed in both the bagasse and CNFs, thereby showing that the cellulose structure did not change significantly during the H_3_PO_4_ and H_2_O_2_ treatments [52]. The characteristic absorption peaks at 1250 cm^−1^ (C–O bond vibration of the aryl group), 1508 cm^−1^ (expansion and contraction of the aromatic ring), 1463 cm^−1^ (CH_2_ symmetry bending), and 1730 cm^−1^ (C=O stretching of the acetyl and urate groups of hemicellulose, or the ester bond of carboxyl groups in lignin to fragrant acid and ferulic acid) show that lignin and hemicellulose were clearly observed in the FTIR spectrum of the bagasse and disappeared in that of the CNFs. This indicated that the lignin and hemicellulose were removed during the treatment [53,54,55,56]. The characteristic peaks at 3435–3375 cm^−1^ and 1629 cm^−1^ redshifted to 3496–3444 cm^−1^ and 1642 cm^−1^ in the FTIR spectrum for the conversion from bagasse to CNFs. According to the literature [57,58], these phenomena suggest the conversion of cellulose I to cellulose II during the treatment.

**Figure 7:**
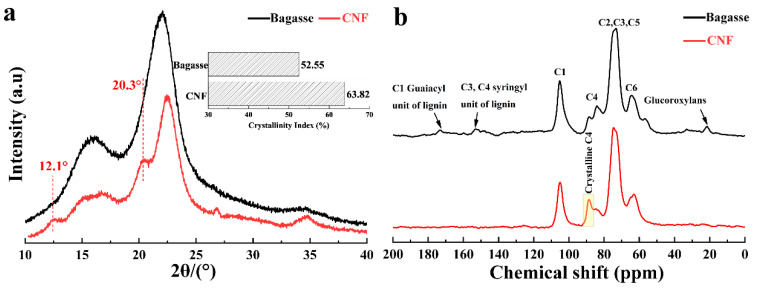
(**a**) X-ray diffraction (XRD) patterns of the bagasse and CNFs. (**b**) ^13^C cross-polarization magic angle spinning (^13^C CPMAS) nuclear magnetic resonance (NMR) spectra of the bagasse fiber and CNFs.

XRD tests were performed to analyze the changes in the crystal form and crystallinity of cellulose for the conversion from bagasse to CNFs, and the experimental results are shown in Figure 7a. As can be seen from Figure 7a, the XRD peaks of the CNFs at 2θ = 16.5° (110), 22.1° (200), and 34.7° (004) for cellulose I were significantly strengthened in comparison with that of the bagasse [59]. The crystallinity of the CNFs and the bagasse was 63.82% and 52.55%, respectively. This result indicated that the proportion of crystalline to amorphous regions in the cellulose was increased due to the removal of amorphous cellulose during the treatment [60]. Additionally, in the XRD patterns of the CNFs, there were clear peaks at 2θ = 16.5° ($\bar{11}0$ for cellulose I), 20.5° (012 for cellulose I), 12.1° ($\bar{11}0$ for cellulose II), and 20.2° (110 for cellulose II) [61]. The existence of the cellulose II structure indicates that cellulose is dissolved and regenerated by phosphoric acid. The structure of cellulose I in CNFs is still the main ingredient. This indicates that the lattice structure of cellulose does not change dramatically during the treatment.

The solid-state ^13^C CPMAS NMR spectra of the bagasse and CNFs are shown in Figure 7b. The important resonance peaks in the NMR spectra of the bagasse and CNFs are summarized in Table 2 [62]. The peaks at ~21 and ~57 ppm (the glucuronic acid of hemicellulose) and the peaks at ~152 and ~173 ppm (the lignin guaiac-based C1 carbon and violet C4 and C3 carbons of syringyl) were clearly observed in the NMR spectrum of the bagasse, but not in that of the CNFs [63], which was attributed to the fact that the lignin and hemicellulose were removed during the treatment. The intensity of the crystalline peak at ~88 ppm increased, while the intensity of the amorphous peak at ~84 ppm decreased, for the conversion from bagasse to CNFs. The crystalline peak at ~88 ppm replaced the amorphous peak at ~84 ppm to become the main peak. These results indicated that the crystallinity of the CNFs was significantly increased in comparison with that of bagasse, which was consistent with the results of the XRD tests.

**Figure 8:**
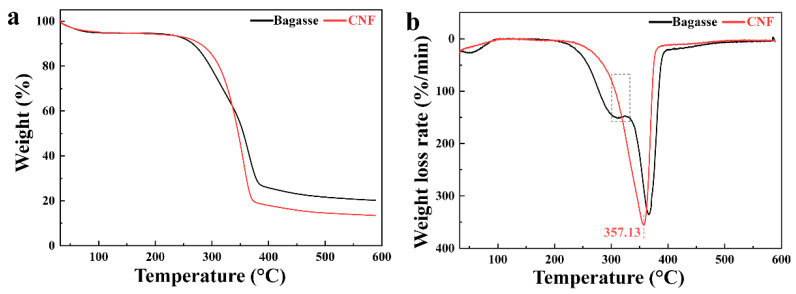
Thermogravimetric analysis (TGA) and DTG curves of the bagasse and CNFs: (**a**) TGA and (**b**) DTG.

Thermal stability is a very important parameter of CNFs. TGA was performed on the bagasse and CNFs, and their TG and DTG curves were compared, as shown in Figure 8. As can be seen from Figure 8a, the residual mass of the bagasse was higher than that of the CNFs, which was attributed to the higher lignin content in the bagasse [64]. As shown in Figure 8b, the low temperature shoulder at 200–315 °C clearly appeared in the TGA curve of the bagasse and disappeared in that of the CNFs because the hemicellulose had been removed in the treatment [65,66]. The maximum decomposition temperature of the CNFs was 357.13 °C, which indicated that the CNFs had a good thermal stability.

## 4. Conclusions

The direct extraction of CNFs from untreated biomass with a compact and complex hierarchy is both important and challenging. The chemical compositions and DP values of the bagasse after treatment in the 72 wt. % H_3_PO_4_ aqueous solution with 30 and 60 mmol/g H_2_O_2_ at 5 and 35 °C for different times (18, 24, 36, and 96 h) were investigated. The CNFs were characterized using TEM, XRD, FTIR, and TGA, and the results were as follows. Due to the cooperative ternary mechanism that consisted of intercrystalline swelling, oxidation, and acid hydrolysis, the lignin and hemicellulose were effectively removed, the plant cell walls were deconstructed, and the cellulose was fibrillated to a 7–22 nm width. Then, a high yield of CNFs with a 12 nm width, 64% crystallinity, and thermal stability was extracted from the untreated bagasse in one bath treatment with H_3_PO_4_ and H_2_O_2_. The CNF yields could reach 99% and 64% based on the initial amounts of cellulose in biomass and the initial amounts of biomass, respectively. This method applies simple steps and inexpensive and easy-to-obtain chemical reagents. Therefore, the current work suggests a simple and cost-effective strategy for producing CNFs.

## Figures and Tables

**Table 1:** Bagasse was treated with time, temperature, and dosage of H_2_O_2_, as well as the yield, chemical component, and removal ratio.

S.N.	Time (h/°C)	H_2_O_2_ (mmol/g)	Yield (%) ^a^	Cellulose	Hemicellulose		Lignin	
				Content (%)	Content (%)	Removal (%)	Content (%)	Removal (%)
		Bagasse		46.50 ± 1.25	27.69 ± 0.84		21.42 ± 1.31	
1	18/35	60	49.75/93.36	87.36 ± 1.14	3.44 ± 0.25	96.32	7.47 ± 0.87	82.65
2	24/35	60	42.21/85.62	94.32 ± 0.61	1.32 ± 0.34	98.80	3.11 ± 0.55	93.87
3	36/35	60	33.95/69.48	95.17 ± 0.32	0.07 ± 0.04	99.95	2.53 ± 0.47	95.99
4	24/35	30	58.30/93.22	74.35 ± 0.96	4.13 ± 0.53	91.18	20.33 ± 0.83	44.53
5	96/5	60	63.75/99.42	72.39 ± 0.74	12.73 ± 0.23	70.69	3.61 ± 0.67	88.73

^a^ Yield based on the initial amount of biomass/yield based on the initial amount of cellulose in biomass.

**Table 2:** Resonance distribution of the main peaks in the ^13^C CPMAS NMR spectra of the bagasse fiber and CNFs.

	Chemical Shift (ppm)	
	Bagasse	CNFs
C1	105.22	105.09
Crystalline C4	88.30 (very small)	88.59 (major)
Amorphous C4	84.11	84.68
Crystalline C6	64.42	63.03
Amorphous C6	64.89	63.19

## Supplementary Materials

The following are available online at https://www.mdpi.com/2079-4991/10/11/2227/s1. Figure S1. AFM 3D image of CNF samples; Figure S2. (a) TEM image of the CNFs; (b) diameter distribution of the CNFs (AD: average diameter); Figure S3. A TEM diagram of the bagasse treated in the 85 wt. % H_3_PO_4_ aqueous solution for 24 h; Figure S4. XRD patterns of the bagasse treated in the 85 wt. % H_3_PO_4_ aqueous solution for 24 h.
