# Supplementary material for: Preparation of Cellulose Nanofibers from Bagasse by Phosphoric Acid and Hydrogen Peroxide Enables Fibrillation via a Swelling, Hydrolysis, and Oxidation Cooperative Mechanism
Source: Nanomaterials (Basel). 2020 Nov 10;10(11):2227. doi: 10.3390/nano10112227 (PMC7696933; doi:10.3390/nano10112227)
Supplement: Supplementary file 1 [file nanomaterials-10-02227-s001.pdf]

nanomaterials

Supplementary information

Preparation of Cellulose Nanofibers from Bagasse by Phosphoric Acid and Hydrogen Peroxide Enables Fibrillation via a Swelling, Hydrolysis, and Oxidation Cooperative Mechanism

Jinlong Wang [1,2], Qi Wang [1,2], Yiting Wu [1,2], Feitian Bai [1,2], Haiqi Wang [1,2], Shurun Si [1,2], Yongfeng Lu [1,2], Xusheng Li [1,2,]* and Shuangfei Wang [1,2]

1 School of Light Industrial and Food Engineering, Guangxi University, Nanning 530004, China; long05360525@163.com (J.W.); w1497417327@163.com (Q.W.); ww1031327514@163.com (Y.W.); baifeitian123@163.com (F.B.); m15305371443@163.com (H.W.); ssr9979@163.com (S.S.); lyf1102891715@163.com (Y.L.); wangsf@gxu.edu.cn (S.W.)

2 Guangxi Key Laboratory of Clean Pulp & Papermaking and Pollution Control, Nanning 530004, China

* Correspondence: lixusheng@gxu.edu.cn; Tel.: +86-0771-3237-301

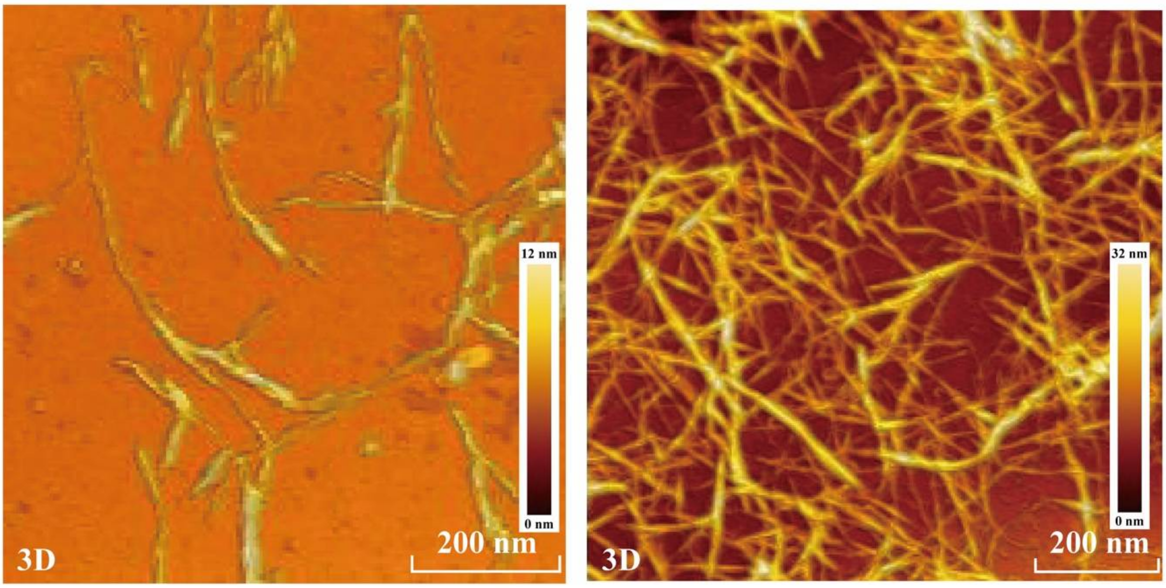

Figure S1. AFM 3D image of CNF samples.

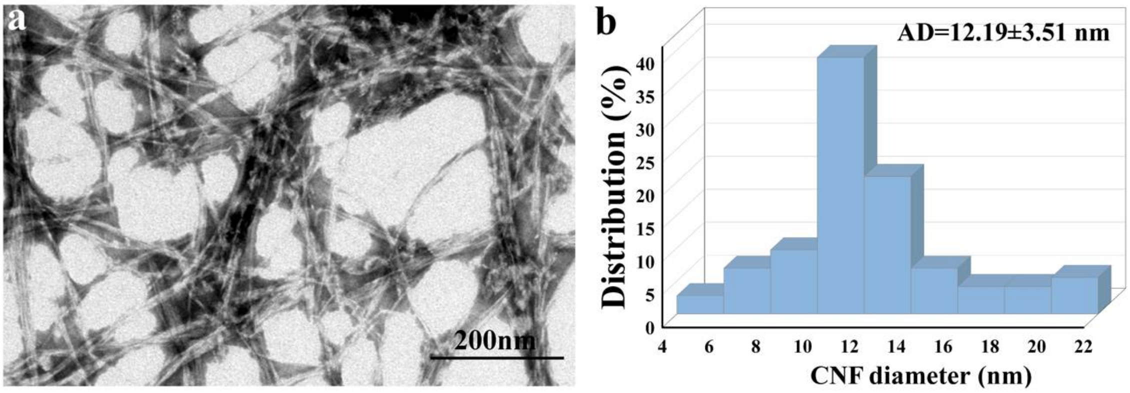

Figure S2. (a) TEM image of the CNFs; (b) diameter distribution of the CNFs (AD: average diameter).

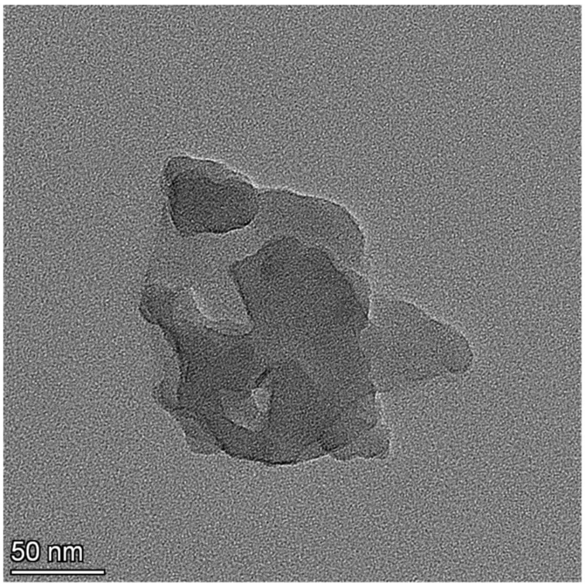

Figure S3. The TEM diagram of the bagasse treated in the 85 wt. % H_3PO_4 aqueous solution for 24 h.

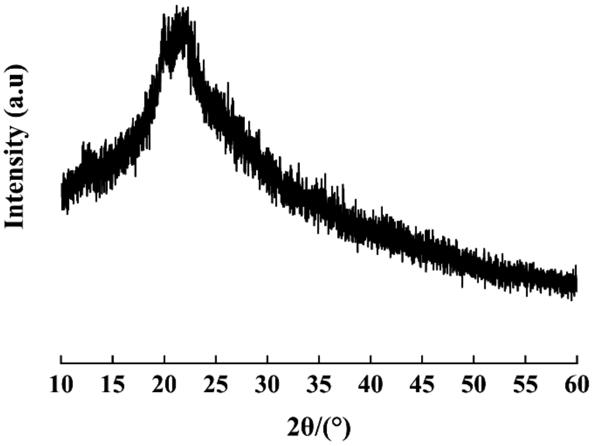

Figure S4. The XRD patterns of the bagasse treated in the 85 wt.% H_3PO_4 aqueous solution for 24 h.

The TEM diagram and the XRD patterns of the bagasse treated in the 85 wt.% H_3PO_4 aqueous solution for 24 h are presented in Figures S3 and S4, respectively. Independent individual fibers were not observed in the XRD patterns (Figure S3). The characteristic absorption peaks at 2θ = 16.5° (110), 22.1° (200), and 34.7° (004) for cellulose I disappear in the XRD spectra (Figure S4). These phenomena indicate that the intercrystalline swelling and intracrystalline swelling of cellulose are related to the concentration of H_3PO_4 solution [1]. The concentration range of H_3PO_4 aqueous solutions that have been shown to cause cellulose to be dissolved is above 77% [2],which should be avoided for the extraction of these CNFs.

References

1. Zhang, Y.-H.P.; Cui, J.; Lynd, L.R.; Kuang, L.R. A transition from cellulose swelling to cellulose dissolution by o-phosphoric acid: evidence from enzymatic hydrolysis and supramolecular structure. *Biomacromolecules* **2006**, *7*, 644-648.
2. Yang, S. Plant fiber chemistry. *Edn* **2001**, *3*, 18-19.
